# Omental Herniation: A Rare Complication of Vacuum-Assisted Closure of Infected Sternotomy Wound

**DOI:** 10.1155/2012/905162

**Published:** 2012-02-26

**Authors:** Philemon Gukop, Madhan Kumar Kuppuswamy, Antonios Kourliouros, Venkatachalam Chandrasekaran

**Affiliations:** Department of Cardiothoracic Surgery, St George's Hospital, London SW17 0QT, UK

## Abstract

Vacuum-assisted closure (VAC) has recently been adopted as an acceptable modality for management of sternotomy wound infections. Although generally efficacious, the use of negative pressure devices has been associated with complications such as bleeding, retention of sponge, and empyema. We report the first case of greater omental hernia as a rare complication of vacuum-assisted closure of sternal wound infection following coronary artery bypass grafting.

## 1. Case Report

**Figure 1:**
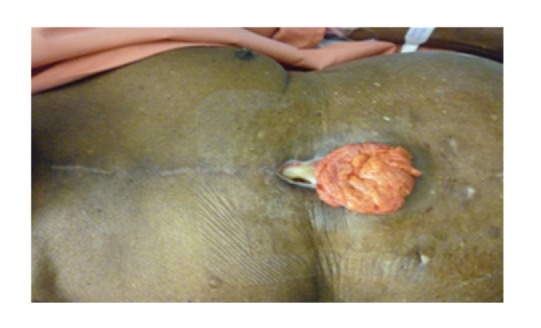
Shows herniated greater omentum through dehisced sternotomy wound after 10 days of VAC application.

A 73-year-old man with comorbidities of noninsulin-dependent diabetes, renal impairment, gout, and poor left ventricular function underwent elective coronary artery bypass surgery for severe three-vessel disease. He made an uneventful recovery and was discharged home on the 7th postoperative day. He was readmitted to hospital 3 weeks later with shortness of breath and was found to have bilateral pleural effusions and bipedal oedema. He was treated with diuretics and had chest tube thoracostomy for effusion drainage. During his admission, the lower sternal and leg wounds were noted to have dehisced. The dehiscence involved the lower 6 cm of the sternotomy wound but there was no clinical evidence of sternal instability. Wound swab cultures grew *Serratia* species. Computed tomography of the chest excluded sternal osteomyelitis. He was commenced on antibiotics as advised by the microbiologist. As there was excessive sanguinopurulent discharge from the sternotomy wound, a vacuum-assisted closure (VAC) device was applied at its lower aspect at a negative pressure of 120 mmHg, and this was changed every 2–3 days. Ten days later, while changing the VAC dressing, a hernia of soft tissue mass consistent with greater omentum was found in the wound measuring approximately 6 cm × 5 cm (Figure 1). The omentum looked pink and healthy but irreducible by gentle manipulation on the ward. As a result of the VAC application at this point, the wound was much smaller and clean with healthy granulation tissue. Further VAC dressing was abandoned and the patient was taken back to the operating theatre where the omentum was reduced under general anaesthetic and the 3-4 cm defect in the linea alba was repaired with interrupted polypropylene sutures. The distal sternal wire was also removed and a size 12Ch Redivac drain was left in the wound for one week. This drained initially about 500–700 mL of serosanguinous fluid per day but subsequently settled. Nylon sutures were removed in 10 days with good results. At 6-month followup, patient was doing well and the wound had healed completely.

## 2. Discussion

Sternal wound infection has an incidence of 1–3% with an associated mortality of 10–25% [1]. Independent risk factors for deep sternal wound infection include obesity, use of bilateral internal thoracic arteries, duration of surgery, postoperative use of inotropic support, and reoperation [2]. Treatment of sternal wound infections includes debridement of the wound until it is clean enough for definitive surgical closure (with or without sternal rewiring), by delayed primary closure, or flap reconstruction by plastic surgeons. VAC is a negative pressure device that aids speedy and nonsurgical debridement of an infected wound by aspirating wound debris/discharge, reducing bacterial colonisation, increasing blood supply to the wound, and enhancing healing by formation of granulation tissue [3, 4]. This technique has recently been adopted as an acceptable means of managing sternotomy wound infections [5–7]. Reported complications of the VAC technique include pain, retention of sponges [8], empyema [9], and the rare but catastrophic event of right ventricular rupture [10]. To our knowledge, there is no report in the literature of herniation of the greater omentum as a result of application of VAC dressing to an infected sternotomy wound. Sternotomy incisions often extend to the upper abdomen and negative pressure of the VAC could aspirate abdominal contents and result in their herniation into the sternotomy wound. Although the greater omentum has capacity to migrate to any site of inflammation on its own accord, the fact that this did not occur until the application of the VAC dressing strongly implicates the VAC as the cause of omental herniation. Such an occurrence contra-indicated further VAC application and necessitates the need for surgical reduction and repair of the defect, which in our case was small with the potential to cause strangulation and ischaemic necrosis of the herniated omentum.

It is possible that the omentum herniated through a defect in the rectus sheath that was inadvertently created during distal dissection of the sternotomy wound, causing communication with the peritoneal cavity. We would have normally performed primary closure for an obvious defect but it is possible that in this case the defect was small and remained unrecognised. Negative pressure (from the VAC pump) may have led to gradual enlargement of this weak point and subsequent herniation. A recent study has shown that cardiac failure, hypertension, diabetes mellitus, coronary artery disease, and renal failure are independent risk factors for sternal wound complications [11]. Our patient presented with florid tissue edema as a consequence of cardiac failure. Tissue edema in the early postoperative period is a substrate for wound dehiscence and infection [11]. Optimal cardiac function is essential for tissue oxygenation and transport of the nutrients/body defence factors required for wound healing and prevention of infection.

Preoperative left ventricular ejection fraction (LVEF) was estimated at 30–35%, while at 3 month followup postoperative LVEF improved to 40–45%. Early and aggressive management of congestive cardiac failure could be preventive of postoperative wound dehiscence and infection. We recommend application of lower and intermittent VAC dressing pressures (<120 mmHg) in infected sternotomy wounds, especially those involving the lower aspects, to prevent this complication. Sternotomy incisions should not extend to the upper abdomen where possible especially in patients with risk factors for poor wound healing/infection.

## 3. Conclusion

Herniation of greater omentum through an infected sternotomy wound is a potentially avoidable rare complication of application of VAC dressing. This contraindicates further application and requires prompt surgical repair. Early optimisation of cardiac failure could reduce postoperative wound dehiscence and infection.
